# The impact of physical activity on the quality of life of a sample of Italian people with physical disability

**DOI:** 10.3389/fspor.2022.884074

**Published:** 2022-10-13

**Authors:** Alessia Biagini, Luca Bastiani, Laura Sebastiani

**Affiliations:** ^1^Department of Translational Research and New Technologies in Medicine and Surgery, University of Pisa, Pisa, Italy; ^2^Epidemiology and Health Research Laboratory, National Research Council, Institute of Clinical Physiology, Pisa, Italy

**Keywords:** physical activity, physical disability, wellbeing, life quality, satisfaction for life

## Abstract

Physical activity promotes psychophysical health; however, to date, only few studies have investigated the association between regular physical activity and the quality-of-life perception and satisfaction in disabled persons. Our aim was to compare the quality and satisfaction for life, self-efficacy, and personal wellbeing of two samples of Italian people with physical disability (amputation, spinal cord dysfunction, neurological disability): one group with people who practiced regular physical activity (active group, *n* = 33) and the other group consisting of sedentary individuals (inactive group, *n* = 26). We compared the mean scores of the groups in the World Health Organization Quality of Life Questionnaire (WHOQoL-BRIEF), the Personal Wellbeing Index, and the Satisfaction With Life and General Self-Efficacy scales. We also compared the impact of rehabilitation, sport, family support, income, job/school, and raising a family on social life and life quality by means of separate MANOVAs. Results of the WHOQoL showed better quality of life in the active than in the inactive group (overall QOL, 4.09 ± 0.7 vs. 3.50 ± 0.9; psychological domain, 72.09 ± 12.7 vs. 62 ± 21.6; social relationships domain, 76.54 ± 16.4 vs. 59.52 ± 24.2). No difference was found for satisfaction with health and life, personal wellbeing, and self-efficacy. The impact of sport on social life and quality of life was greater in active than in inactive individuals. Findings suggest positive effects of physical activity on the perception of quality of life in disabled people. However, they do not allow disentangling whether physical activity is practiced by patients with good quality of life or whether physical activity is responsible for better quality of life.

## Introduction

Physical activity may have a positive impact on psychophysical health. In fact, it may contribute to improving the functioning of the cardio-respiratory system; strengthening of bones, muscles, and articulation; and the maintaining healthy body weight, thus playing an important role in reducing the risk of and preventing chronic pathologies (1). Moreover, there is evidence in the literature that suggests that physical activity may have positive effects on one's body perception, self-esteem, and mood and may offer a social context that promotes the construction of strong social relationships (2).

To date, it is widely accepted that physical activity promotes the psychophysical health of persons with disabilities (3–7). In fact, regular recreational activity and sports participation have been demonstrated to be positively associated with improvements in quality of life, life satisfaction, community reintegration, mood, and employment in individuals with disabilities (8).

In particular, sports can help reduce the discrimination associated with disability by offering a positive context in which the interaction between persons with and without disabilities can develop. Moreover, adapted sports, in addition to facilitating social contacts, help individuals with physical disabilities to become physically and mentally stronger and develop greater independence, by focusing on their abilities, rather than disabilities (8).

Although the importance of sport for physical and psychological wellbeing is a widely shared concept, physical inactivity is largely common in people with disability. For instance, it has been found that persons with acquired brain injuries are inclined to a sedentary way of life, and this increases the risk of secondary health complications (9) such as cognitive, sensorimotor, behavioral, and social problems that can impair the quality of life (10). In the United States, it has been estimated that about 50% of adults with disability are physically inactive and have a greater probability of having a chronic disease (11). According to the Italian National Institute of Statistics (ISTAT), in the Italian population, the percentage of people with bodily limitations who engage in some type of physical activity is low, with the lowest percentages for individuals with severe physical disabilities (12). Actually, in Italy, physical inactivity is an important issue also among healthy people. In fact, about 53% of the population does not meet the guidelines of the World Health Organization for moderate physical activity (13). The reasons behind the lack of physical activity are various, but some of them can be attributed to the suboptimal urban design (i.e., shortage of green spaces/walkable areas and of efficient and cheap public transportation) of many Italian cities. In addition to this, the “accessibility” is often one of the main problems for people with disability, being many of the Italian gyms not adequately equipped with the aids necessary for people with physical disability (13).

To date, only few studies have investigated the association of physical activity with the enhancement of quality of life and life satisfaction of persons with disabilities, and no systematic studies have been carried out in the Italian population (3, 14–18). The aim of the present study was to evaluate the quality of life, satisfaction for life, self-efficacy, and personal wellbeing in a sample of Italian people with physical disability and to compare the scores of the individuals who practice regular physical activity with those of a sample of sedentary individuals. Based on the assumption that physical activity is an important factor in promoting a better quality of life and wellbeing also for people with physical disability, our hypothesis is that among them, people engaged in a regular physical activity could report higher scores in the aforementioned dimensions.

## Methods

### Design

The study consists of a cross-sectional study evaluating the quality of life, satisfaction for life, self-efficacy, and personal wellbeing in two samples of Italian people with physical disability: one group comprising people practiced regular physical activity (active group) and the other one consists of sedentary individuals (inactive group).

### Participants

G^*^Power 3.1.9.7 was used to calculate the required sample size: with α = 0.05, effect size = 0.3, and power = 0.85, the minimum required total sample size for MANOVA with two groups and six response variables was 58. People (*n* = 59; males = 22; females = 33) who agreed to take part in the research were aged between 18 and 60 (mean age ± SD, 35.44 ± 12.32) years. They were recruited at the Cisanello Hospital of Pisa and territorial rehabilitation services as well as through the USID service of the University of Pisa, which sent an email to the people with the characteristics sought for the sample. People were included in the study if they were ≥ 18 and < 60 years of age and had a physical disability (amputation, spinal cord dysfunction, or neurological disability) congenital or acquired before the age of 10. The absence of overt psychiatric disorders was assessed through the Self-Report Symptom Inventory-Revised (SCL-90-R) (19) and STAI-Y2 (20).

This study was approved by the Committee on Bioethics of the University of Pisa (Review No. 31/2019) and the participants signed the informed consent to participate in the study.

### Research instruments and procedure

All the participants filled out a series of questionnaires in a single session, either online or in person. Questionnaires consisted of a general personal data questionnaire and the Italian versions of the WHOQoL-BRIEF (World Health Organization Quality of Life assessment) (21), Satisfaction With Life Scale (SWLS) (22), Personal Wellbeing Index (PWI) (23), and General Self-Efficacy Scale (GSE) (24). The general personal data questionnaire included questions on demographic variables (age, sex, education level, job, type of disability, and pharmacological therapies) and relative to participants' physical activities (e.g., type of physical activity practiced, how tiring it is to practice it, and how many times a week it is practiced). Moreover, the participants were asked to indicate on a 7-point Likert scale (from 1, not important, to 7, extremely important) how important rehabilitation, physical activity, family support, income, job/school, and raising a family were for their social life and life quality.

The WHOQoL-BREF is a quality-of-life assessment, based on a 26-item self-report scale, which is cross-culturally applicable. The WHOQoL-BREF is strictly related to the individual psychological and physical statuses but, at the same time, allows a subjective evaluation of the role of social environment in the perceived wellbeing. In fact, the WHOQoL-BREF measures four domains related to the quality-of-life (QoL) construct, namely, physical health, psychological, social relationships, and environment domains, plus overall QoL (item 1: “How would you rate you QOL?”) and general satisfaction with health (item 2: “How satisfied are you with your health?”). The scale is measured on a 5-point Likert scale, with higher scores indicating a higher self-perception of the QoL.

The SWLS is a 5-item self-evaluation scale designed to measure global cognitive judgments of one's life satisfaction; the participants were required to indicate on a 7-point Likert scale how much they agree or disagree with each of the five items (from 1, strongly disagree, to 7, strongly agree).

The PWI scale is a self-evaluation scale that gives a domain-level representation of subjective global life satisfaction. The core set of items comprises seven questions of satisfaction with specific life domains: standard of living, personal health, achieving in life, personal relationships, personal safety, community connectedness, and future security; and two additional (optional) items refer to general life satisfaction (“Thinking about your own life and personal circumstances, how satisfied are you with your life as a whole?”) and satisfaction with religion or spirituality (“How satisfied are you with your spirituality or religion?”). The scores of the core set of items plus the religion item, if answered, are averaged to produce a Personal Wellbeing Index. The participants were required to indicate on a 10-point Likert scale how much satisfied they are (from 0, very unsatisfied, to 10, very satisfied). The item relative to satisfaction with life as a whole is not a component of the PWI and is analyzed as a separate variable and generally used to test the construct validity of the PWI. The scores relative to the optional item on satisfaction with religion or spirituality were also analyzed separately. In fact, data derived on the PWI scale items may be used either aggregated and averaged or at the level of individual domains.

The General Self-Efficacy Scale (GSE) is a 10-item self-evaluation scale used to measure self-efficacy. The participants were required to indicate on a 4-point Likert scale how much they agree or disagree with each of the items (from 1, strongly disagree, to 4, strongly agree).

### Statistical analysis

Participants who practiced physical activity for at least 2–3 times a week were assigned to the active group, while participants not engaged in any regular physical activity to the inactive group.

Percentage scores relative to the general personal data questionnaire were compared between the two groups by means of Pearson chi square. A *t*-test was also used to evaluate differences in age. Homogeneity of variances was checked by means of Levene's test.

The impact of rehabilitation, sport, family support, income, job/school, and raising a family on social life and life quality of the two groups was analyzed by means of two separate multivariate ANOVAs (MANOVAs) with group (active, inactive) and sex (male, female) as independent variables, and the scores on the various items as dependent variables.

We compared the mean scores of the two groups in the WHOQoL-BRIEF by means of MANOVA, with group (active, inactive) as independent variables, and items 1 and 2 and the four domains (physical health, psychological, social relationships, and environment) as dependent variables. A separate MANOVA, with group (active, inactive) as independent variable was also performed to compare the global scores of the two groups in SWLS, PWI, and GSE (dependent variables). For each scale, the item internal consistency was assessed by means of Cronbach's alpha. Significance was set at *p* < 0.05. The SPSS.15 statistical package was used for all analyses.

## Results

People who agreed to take part in the research were 59 (males = 22; females = 33) and had a mean age of 35.44 ±12.32 years (mean ± SD). A total of 33 participants were assigned to the active group, while 26 participants to the inactive group. The type of physical activity carried out by the active participants was various (fencing, swimming, soccer, archery, gym, table tennis, walking, CrossFit, kayak). In total, 27 participants of the active group (81.8%) performed a sport at the competitive level (e.g., national competitions).

All the participants took <30 min to complete the forms (mean time: 10.97 ± 6.33); two tetraplegic participants of the active group reported receiving some help in compiling the questionnaires.

Table 1 shows the demographic data of the two groups of participants. No significant difference (df = 48.84, *t* = −1.82, *p* = 0.075) was found between the mean age of participants belonging to the inactive (mean ± SD, 38.77 ± 13.76) and the active group (32.82 ± 10.55). Moreover, Pearson chi square analysis showed no differences between the two groups for job (χ^2^ = 5.33; *p* = 0.07), education (χ^2^ = 1.48; *p* = 0.48), type of disability (χ^2^ = 0.85; *p* = 0.65), ability to walk (χ^2^ = 0.54; *p* = 0.76), and use of pharmacological treatments (χ^2^ = 0.76; *p* = 0.38).

**Table 1:** Demographic data.

	**Active**		**Inactive**
	***N* = 33**		***N* = 26**
**Age**	32.82 ±10.55		38.77 ± 13.76
**Sex**			
F	15 (45.5%)		18 (69.2%)
M	18 (54.5%)		8 (30.8%)
**Education level**			
Middle school diploma	2 (6.1%)		4 (15.3%)
High school diploma	21 (63.6%)		14 (53.8%)
Degree	10 (30.3%)		8 (30.8%)
**Job**			
Employed	14 (42.4%)		4 (15.4%)
Unemployed	16 (48.5%)		17 (65.4%)
Retired	3 (9.1%)		5 (19.2%)
**Disability**			
Amputation	9 (27.3%)		10 (38.5%)
Spinal cord dysfunction	13 (39.4%)		9 (34.6%)
Neurological	11 (33.3%)		7 (26.9%)
**Ability to walk**			
No	9 (27.3%)		9 (34.6%)
Yes	10 (30.3%)		6 (23.1%)
Yes, with aids	14 (42.4%)		11 (42.3%)
**Continuous pharmacological treatment**			
No	24 (72.7 %)		13 (50 %)
Yes	9 (27.3 %)		13 (50 %)

**Figure 1:**
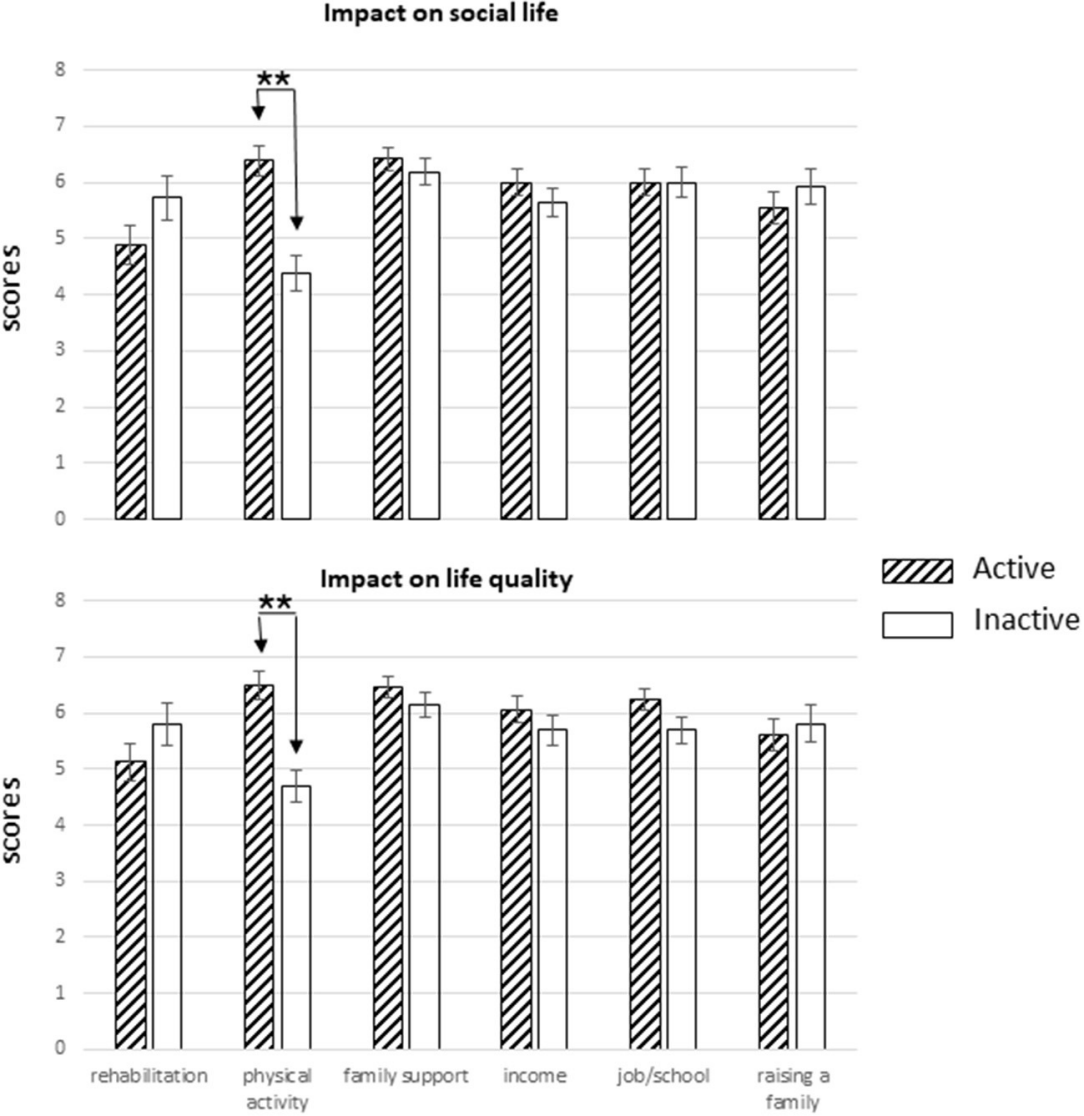
Impact of rehabilitation, physical activity, family support, income, job/school, and raising a family on social life **(upper panel)** and life quality **(lower panel)** of active and inactive groups. For each group, the mean score (± SEM) of each variable is shown. Significant differences (**p < 0.01) between the groups are indicated.

Considering the impact of rehabilitation, physical activity, family support, income, job/school, and raising a family on social life and quality of life, MANOVA showed significant group effects for both social life [*F*_(6,52)_ = 4.91, *p* < 0.0005, η^2^ = 0.362, power = 0.98] and quality of life [*F*_(6,52)_ = 4.23, *p* < 0.002, η^2^ = 0.328, power = 0.96]. In particular, the two groups have different scores only for the physical activity item [social life, *F*_(1,57)_ = 24.24, *p* < 0.0001; quality of life, *F*_(1,57)_ = 21.87, *p* < 0.0001] with higher scores in the active than in the inactive group (Figure 1, upper and lower panels).

Table 2 shows the mean scores (± SD) of the two groups in the first two items (item 1: overall QOL; item 2: general satisfaction with health) and in the four domains of the WHOQoL (Cronbach α = 0.805). MANOVA yielded a significant group effect [*F*_(6,52)_ = 3.13, *p* = 0.008, η^2^ = 0.28, power = 0.90], with significantly higher scores in the active than in the inactive group for item 1 [overall QOL; *F*_(1,57)_ = 7.78, *p* = 0.007] and for the psychological [*F*_(1,57)_ = 5.01, *p* = 0.029] and social relationships [*F*_(1,57)_ = 6.78, *p* = 0.012] domains. An almost significant difference was found for the physical domain [*F*_(1,57)_ = 3.73, *p* = 0.059].

**Table 2:** WHOQoL, GSE, PWI, and SWSL scores.

	**Active**		**Inactive**	
	**Mean**	**SD**	**Mean**	**SD**
**WHOQoL**				
QOL rating	4.09^*^	0.72	3.50	0.91
Satisfaction with health	3.72	0.72	3.35	1.02
Physical health	72.75	15.75	64.42	17.33
Psychological	72.09^*^	12.78	62.00	21.56
Social relationships	76.54^*^	16.36	59.52	24.20
Environment	62.97	17.46	59.27	17.55
**GSE**	31.15	5.13	29.19	7.60
**PWI**	7.27	1.60	7.19	1.79
**SWSL**	4.94	1.43	4.50	1.50

Significant differences between groups are indicated,

* p < 0.05.

MANOVA performed on SWLS (α = 0.858), PWI (α = 0.856), and GSE (α = 0.921) did not reveal any significant group effect (Table 2). The item “How satisfied are you with your spirituality or religion?” of the PWI scale was answered by 23 participants of the inactive group and 31 of the active group, and an almost significant difference [*F*_(1,53)_ = 3.61, *p* = 0.06] was found between the mean scores of the two groups (inactive, 6.91 ± 2.74; active group (5.19 ± 3.64).

## Discussion

The present study carried out in a sample of Italian people with physical disabilities (spinal cord dysfunction, limb amputation, neurological disability) was aimed to compare the quality and satisfaction for life, self-efficacy, and personal wellbeing of the individuals who practice regular physical activity with those of sedentary individuals. The two groups were similar in terms of age, type of disability, ability to walk, and pharmacological treatments. No differences were also found in the education and employment levels.

The scales we have employed have been previously used to evaluate the quality-of-life perception and wellbeing of individuals with disability, such as persons with amputations and spinal cord injuries (16–18).

The results of the present study indicate that people with disabilities who practice physical activity report better QoL than people not engaged in any regular physical activity, whereas no significant difference between the groups was found for the satisfaction with the health item. This is particularly interesting because the relation between physical health and psychological wellbeing has been challenged by studies conducted in women with fibromyalgia who may report high wellbeing in the presence of high-intensity, high-extension, and disabling musculoskeletal pain (25). The results of the single domains indicated that social relationships and psychological scores were different between the two groups, with higher scores in the active group than in the inactive group.

The lack of differences in the environment domain is not surprising as it primarily consists of financial resources, independence, physical safety and security, accessibility and quality of health and social care, the home environment, and transport, all these factors being similar in the two groups.

Findings confirm that physical activity has positive effects on the perception of quality of life and suggest that physical activity, in general, offers an important chance to establish and strengthen social relationships for people with disabilities. Moreover, higher scores in the psychological domain suggest that active people have a better control on their emotions and a better relation with their own body image and look and higher self-esteem levels than inactive people. It is likely that performing physical activity could, in fact, help individuals to be more focused on their capabilities, rather than disabilities, thus improving the way they cope with aversive situations and in particular with their disability.

Our findings are partially in accord with a previous study performed in a sample of people with amputation or paraplegia (18), which showed that people who participated in adapted sports had significantly higher QoL and life satisfaction scores than people with physical disabilities not involved in any adapted sports. In this study, significant differences between physically active and sedentary individuals were found in the physical domain. By contrast, in the present study, the scores in the physical domain of active people tend to be higher than those of inactive individuals, but this difference does not reach statistical significance.

Our study also shows that in the active group, participation in physical activities was the most relevant factor, together with family support and job/school, that affected QoL and satisfaction with health, while in the inactive group, in addition to family support, rehabilitation and raising a family were the most important factors related to QoL and health. Physical therapy usually does not continue long term, and we can assume that regular physical activity such as adapted sports can actually be considered as a sort of replacement of formal rehabilitation. Thus, we can assume that people with disabilities who do not practice any adapted physical activity/sports are more likely to try to continue formal rehabilitation than active people.

Although statistical analysis did not yield any difference between the two groups in the other scales (SWLS, PWI, and GSE), an almost significant difference was found for the optional item of the PWI scale relative to satisfaction with one's own spiritual life/religion that was answered by a subsample of participants. In this case, sedentary individuals tend to rely more on religious faith and to be more satisfied with it than physically active people, which suggests that the two groups employ different coping strategies. In fact, we can assume that while for physically active people, physical activity represents a major coping strategy, and for sedentary individuals, religiosity and spirituality may function as important resilience-driving factors (26, 27).

A limitation of the present study is that it does not allow disentangling whether physical activity and QoL are the cause or the effect. Specifically, the question remains whether physical activity is a useful tool to achieve better QoL even in disabled people or whether it is a high QoL score in disabled people that makes them more inclined to practice physical activity. About this, it would be interesting to study whether patients with disabilities practicing other activities (i.e., playing music) may report better quality of life with respect to patients not reporting satisfying activities and/or hobbies (28, 29). Another limitation is that we enrolled patients independently from the duration of their disability, which could influence the construction of their personality, including self-efficacy. Finally, a larger sample of participants could provide statistically stronger results.

Further research should assess whether personality characteristics such as anxiety, depression, alexithymia, locus of control, type of disability, and the presence of pain may influence the association between sport activity and quality of life in patients with disabilities (30).

## Conclusion

In conclusion, present findings suggest positive effects of physical activity on the perception of quality of life in people with disabilities. Thus, we think that policies that promote wellbeing through active lifestyles should increasingly include the improvement of accessibility to physical activity for people with disabilities.

## Data availability statement

The raw data supporting the conclusions of this article will be made available by the authors, without undue reservation.

## Ethics statement

The studies involving human participants were reviewed and approved by Committee on Bioethics of the University of Pisa (Review No. 31/2019). The patients/participants provided their written informed consent to participate in this study.

## Author contributions

LS and AB conceived and coordinated the study. AB carried out the study. LB, LS, and AB performed the statistical analysis and reviewed and edited the manuscript. LS drafted the manuscript. All authors have read and approved the final version of the manuscript and agree with the order of presentation of the authors.

## Funding

This work was supported by the University of Pisa (Ateneo 2021).

## Conflict of interest

The authors declare that the research was conducted in the absence of any commercial or financial relationships that could be construed as a potential conflict of interest.

## Publisher's note

All claims expressed in this article are solely those of the authors and do not necessarily represent those of their affiliated organizations, or those of the publisher, the editors and the reviewers. Any product that may be evaluated in this article, or claim that may be made by its manufacturer, is not guaranteed or endorsed by the publisher.
